# Feline Peritoneal Effusions—A Poor Prognosis?

**DOI:** 10.3390/ani15223355

**Published:** 2025-11-20

**Authors:** Laura Letwin, Sivert Nerhagen, Camilla Hindar, Barbara Glanemann

**Affiliations:** 1 AURA Veterinary, 70 Priestley Road, Guildford GU27AJ, UK; 2 Department of Companion Animal Clinical Sciences, Faculty of Veterinary Medicine, Norwegian University of Life Sciences, 1432 Ås, Norway; 3 Queen Mother Hospital for Animals, Royal Veterinary Collage, Hatfield AL97TA, UK

**Keywords:** ascites, survival, cat

## Abstract

Feline ascites has been reported to have a poor prognosis, with a median survival time of 21 days (considering all etiologies). However, previous studies included relatively small populations (<100 cases) and there is no literature evaluating the prognosis of all causes of feline ascites within the last 20 years. This study aimed to assess the survival times of a large population of cats presenting with ascites and assess the effect of the effusion cause, signalment, clinicopathological and imaging findings on survival. Data was acquired from the medical record system of a referral hospital (including both referrals and first-opinion emergency cases). Four hundred and ninety-eight cats met the inclusion criteria and 55% of all cases survived to discharge. Median survival time post-discharge was 30.5 days. The cause of the effusion was significantly associated with survival to discharge (*p* = 0.002). Common etiologies of ascites included neoplasia, septic peritonitis, sterile inflammatory disease, uroperitoneum, hemoperitoneum and cardiac disease. Uroperitoneum cases had the highest rate of survival to discharge (77%), while hemoperitoneum cases had the lowest percentage surviving to discharge (40%). Subjectively assessed effusion volume on imaging was significantly associated with survival to discharge (*p* = 0.012). Subjective assessment of the effusion volume and effusion assessment via abdominocentesis to help obtain a diagnosis can help guide prognosis and are diagnostics that do not require advanced techniques or specialist equipment, which can provide important prognostic information for cats presenting with ascites.

## Simple Summary

Feline ascites, an accumulation of fluid in the abdominal cavity, can be caused by many different underlying disease processes. The aim of this study was to provide information on the prognosis associated with this finding and help guide first-opinion practitioners to make further decisions for feline patients that are found to have ascites. We found that 55% of cases with ascites survived to discharge from hospital with an average survival time of 30.5 days after discharge. Some types of abdominal fluid were also found to be associated with a higher rate of survival to discharge, with 77% of cases with urine in the abdominal cavity (uroabdomen) surviving to discharge. Conversely, cases with blood in the abdominal fluid (hemoperitoneum) and had a lower chance of surviving to discharge (40%). This is important information, as the type of abdominal fluid can be determined using relatively simple and inexpensive diagnostic tests and can be used to help guide the prognosis of patients and their families on possible outcomes.

## 1. Introduction

Ascites is an accumulation of fluid in the abdomen, indicating an underlying pathology. Ascites has previously been reported as a relatively uncommon clinical presentation in cats within referral hospitals, with an incidence rate of 3 per 1000 cases [1], although prevalence has been speculated to be higher in first-opinion practice [2].

The cause of ascites in humans varies geographically, but it is most commonly caused by portal hypertension, e.g., due to hepatic cirrhosis [3]. Non-portal hypertensive causes include malignant ascites, tuberculosis peritonitis and secondary bacterial peritonitis [4]. The prevalence of different causes of ascites in canine and feline patients is not well described. The pathophysiological causes of ascites are commonly divided into decreased oncotic pressure (e.g., hypoalbuminemia), increased hydrostatic pressure (e.g., congestive cardiac failure and portal hypertension) and increased vascular permeability due to inflammation (e.g., bacterial peritonitis) [5]. Other pathologies which may be caused by more than one pathophysiological mechanism include chylous effusions, hemorrhage, neoplastic effusions and body fluid-related effusions (e.g., bile peritonitis and uroabdomen) [6,7].

Only one previous study has evaluated survival times in 65 cats presenting with ascites regardless of etiology, reporting a median survival time of 21 days [1]. Other small case studies (n = 65 cats and n = 34 cats) looking at survival times in cats with ascites caused by specific etiologies, including hemoperitoneum and exocrine pancreatic carcinomas, have also reported a guarded prognosis [8,9]. This study aimed to assess the survival times of a larger population of cats presenting with ascites. Further objectives of this study were to identify any age, sex or breed associations with the survival of cats with confirmed ascites (based on imaging and the cytological assessment of peritoneal effusion). The study also aimed to determine the prognosis associated with different underlying causes of peritoneal effusions as well as identifying any association between survival and type of effusion.

## 2. Materials and Methods

### 2.1. Data Acquisition

The medical record system of a tertiary referral hospital was searched for cats that were recorded as having an abdominal effusion (including patients seen as both referrals and as emergency first-opinion cases) between January 2004 and June 2020. Search terms used included “peritoneal” or “abdominal” and “ascites” or “effusion”.

Cats were included in the study if they had an abdominal effusion confirmed by imaging and some form of fluid analysis of the effusion (either total protein and total nucleated cell count and/or cytological analysis). Cats were also included without premortem fluid analysis in cases deemed due to congestive heart failure (CHF) based on echocardiography performed by, or supervised by, a board-certified cardiologist (ACVIM or ECVIM), or if the effusion was sampled on postmortem examination. Cats were excluded if imaging was inconclusive for a peritoneal effusion or if the effusion had not been sampled. A total of 2336 cases were excluded due to a lack of effusion sampling, available analysis or confirmation of ascites.

Information regarding signalment, cytological characterization of the effusion (including total protein and total nucleated cell count), imaging performed, diagnostics (including complete blood count, serum biochemistry and any ancillary diagnostic tests) and procedures such as exploratory laparotomy and retroviral testing was collected. A subjective assessment of the size of effusion across all imaging modalities was also recorded where available. This assessment was made by a veterinarian assessing the imaging (this could be a primary care veterinarian working in the first-opinion emergency service of the hospital, an emergency and critical care resident, a clinician or a radiology resident supervised by a board-certified radiologist or a board-certified radiologist) and was not standardized between observers. Cats with an effusion described as scant, mild, minimal, small or another synonym were classified as mild. Similarly, cases described with moderate, severe or equivalent synonyms were grouped into moderate or marked, respectively. If a case was described as having a “mild to moderate” effusion, they were grouped with “moderate”, and if they were described as having a “moderate to marked” effusion, they were grouped with “marked”. If there was disagreement between a point-of-care ultrasound (POCUS) and a more advanced imaging modality, the case was grouped as per the description written or reviewed by a board-certified radiologist.

In cases with available data, the abdominal effusions were defined as a transudate, protein-rich transudate or exudate based on total nucleated cell count (TNCC) and total protein (TP). A transudate was defined as having a TNCC of <1.5 × 10^9^/L with a TP of <20 g/L [5]. A protein-rich transudate was defined as having a TNCC of <5.5 × 10^9^/L and a TP of >20 g/L, while an exudate was defined as having a TNCC of >5 × 10^9^/L and a TP of >20 g/L with the majority of the cells being neutrophils [5,10].

Cases were also characterized based on the causes of the effusion: hemoperitoneum, bile peritonitis, uroperitoneum, idiopathic chylous effusion, neoplastic, cardiogenic, feline infectious peritonitis (FIP), septic peritonitis, trauma, hypoalbuminemia, volume overload, sterile inflammatory disease, recent abdominal surgery, portal hypertension and undefined. In cases where there was overlap between these causes, e.g., a neoplastic mass rupture had caused a septic peritonitis, cases were grouped in the most likely cause of the effusion itself, i.e., septic peritonitis. Similarly, if cases with a uroperitoneum were also found to have a septic effusion, they were still characterized as a uroperitoneum based on the most likely primary cause of the effusion. Hemorrhagic effusions were defined based on a packed cell volume (PCV) of 3% [5,10]. The diagnosis of bile peritonitis was based on serum and fluid biochemistry demonstrating at least twice the concentration of bilirubin in abdominal fluid compared to serum bilirubin or on exploratory laparotomy documenting biliary tract leakage [10,11]. Cases were categorized as having uroperitoneum when abdominal fluid creatinine +/− potassium was found to be higher than serum creatinine and potassium, or when urinary tract perforation had been documented based on contrast imaging. Chylous effusions were defined as effusions with a fluid triglyceride concentration of >1 mmol/L and where the fluid cholesterol/triglyceride ratio was <1 (when converted into mg/dL) [10,12,13]. Cases were also characterized based on the causes of the effusion: hemoperitoneum, bile peritonitis, uroperitoneum, idiopathic chylous effusion, neoplastic, cardiogenic, feline infectious peritonitis (FIP), septic peritonitis, trauma, hypoalbuminemia, volume overload, sterile inflammatory disease, recent abdominal surgery, portal hypertension and undefined.

Survival data was based on information on survival to discharge from hospital records and, where possible, information gained from follow-up visits or by contacting referring practices.

### 2.2. Statistics

Microsoft Excel was used to compile data and summarize descriptive statistics. SPSS IMB software [Version 30.0.0.0 (172)] was also used for descriptive statistics and further statistical testing. A *p* value of <0.05 was considered statistically significant. Categorical comparisons were performed using the chi-square (χ^2^) test. For statistical analysis, breeds were defined as pedigree or non-pedigree. Continuous variables were assessed for normality graphically and using the Shapiro–Wilk test. Associations between categorical and continuous data were compared using the Mann–Whitney U-test for non-normally distributed continuous variables, and the two-sample t-test for normally distributed continuous variables. Multivariable analysis was also used to look for associations between variables and survival to discharge. A Kaplan–Meier curve was used to show median survival times. Cats that were lost to follow-up or that were still alive at the time of data collection were censored. Cases that were lost to follow-up were also excluded from calculations of median survival times. Due to the recent developments in treatment for feline infectious peritonitis (FIP) which has greatly improved the prognosis of these cases, overall statistical analysis was performed with and without FIP cases.

## 3. Results

### 3.1. Signalment

A total of 2834 cases were found in the records search, and 498 cats met the inclusion criteria. The median age was 7 years (range 3 months–8.75 years). Of these, 277 (56%) were neutered male, 29 (6%) entire male, 176 (35%) neutered female and 16 (3%) entire female. The most common breed was the Domestic Short Hair (n = 286 cats) followed by the Domestic Long Hair (n = 40 cats) and the British Short Hair (n = 33 cats). Other common breeds included Bengal (n = 18 cats), Ragdolls (n = 13 cats), British Blue (n = 11 cases) and Persians and Siamese, of which there were 10 cases of each. Overall, 152 cats were defined as pedigree breeds (31%) in the study population. Excluding cases with FIP, only 106/429 (25%) cases were defined as pedigree breeds.

### 3.2. Effusion Analysis

Of the 498 cases, abdominal fluid was sampled in 471 (95%). The type of effusion was able to be defined in 366 cases (73%), while data (either TP, TNCC or a pathology report) was missing in the remainder of cases. Both TP and TNCC were available for 333 cases (67%). Of the cases where the abdominal effusion fluid was not sampled, a diagnosis of cardiac disease was made via echocardiography (n = 22), or a diagnosis was established postmortem (n = 5).

Of the cats in which the type of effusion was able to be defined, a protein-rich transudate was identified in 44% (n = 160) of cats, 30% cats (n = 110) had an exudative effusion and 14% (n = 51) of effusions were defined as a pure transudate. Hemoperitoneum was also identified in 11% (n = 41) of cases with a defined type of effusion, and a chylous effusion was identified in 2% (n = 6), Table 1.

### 3.3. Causes of Ascites

The underlying cause of ascites was identified in 486/498 cats (98%), as detailed in Table 2. The most common diagnosis was septic peritonitis (n = 86, 17%), followed by a neoplastic effusion (n = 84, 17%) and sterile inflammatory causes other than FIP (n = 70, 16%). FIP was diagnosed in 69 cats (14%), cardiac disease was diagnosed in 44 cats (9%), a uroperitoneum was found in 43 cats (9%) and 35 cats (7%) were diagnosed with a hemoperitoneum. Twenty cats were found to have volume overload (4%), eight cats (14%) had an effusion attributable to recent abdominal surgery, five cats (1%) had hypoalbuminemia and four (1%) were diagnosed with bile peritonitis. Each diagnosis of idiopathic chyloabdomen, portal hypertension and trauma was found in three cats (<1%). Twelve cases had an undefined cause of the effusion, e.g., when overlapping etiologies were present or a clear cause had not been identified.

### 3.4. Imaging

All cases had some form of abdominal imaging with point-of-care ultrasound (POCUS) and abdominal ultrasound being the most common imaging modalities used (n = 265 and n = 391, respectively). In total, 394 cats had some form of thoracic imaging (POCUS, echocardiography, thoracic radiographs or thoracic CT scan, or a combination) (Table 3).

The abdominal effusion was described as mild or a synonym across all imaging modalities in 166 (33%) cases; it was described as moderate in 135 (27%) cases and marked in 131 (26%) cases. The amount of effusion was not described in 66 (13%) cases. The cause of the effusion was significantly associated with the volume of effusion (*p* < 0.001); all bile peritonitis cases had a scant effusion, while 30 (48%) FIP cases with an available effusion description had a marked effusion—a higher percentage than in any other category (Table 3). Out of the cases that underwent thoracic imaging (n = 394), a concurrent pleural effusion was identified in 127 (32%) (Table 3). Point-of-care ultrasound scan failed to identify an abdominal effusion in 16% (n = 44) of cases, which was subsequently identified on other imaging modalities or postmortem. In total, 57% (n = 25) of these cases had an effusion described as mild or a synonym, 27% (n = 12) had an effusion described as moderate and 5% (n = 2) had an effusion described as marked, with 6 cases lacking a description of the size of effusion. For the two cases with marked effusion, the effusion was identified on abdominal ultrasound scan the following day in one of the cases, while the other was found postmortem. It seems likely that both had developed during hospitalization.

### 3.5. Survival

**Figure 1:**
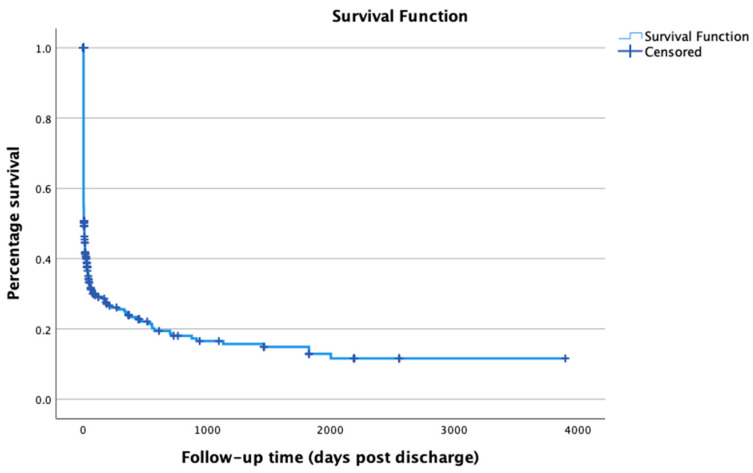
Kaplan–Meier survival curve of survival.

Out of 498 cats, 276 (55%) survived to discharge. For cases that survived to discharge, 108 cases had a defined end point (date of death recorded). The median survival time of cases surviving to discharge was 30.5 days post-discharge, with a range of 1–3900 days. Twelve cases were still alive by the time of data collection (median survival post-discharge was 2190 days with a range of 1095–3900 days). By one-year post-discharge, 45/498 (12.5%) cases that initially presented were known to be still alive, and 139 (28%) cases had been lost to follow-up. Overall, 314 (63%) were dead one year after initial presentation (Table 4 and Figure 1).

Out of the 330 cases that were confirmed to have died by the end of the study, 59 cases died of natural causes (44 prior to discharge), while 261 cases were confirmed to have been euthanized (178 prior to discharge) and 10 cases had an unknown cause of death. Stated causes of euthanasia were due to prognosis, welfare or for financial reasons. The 10 cases with an unknown cause of death were all cases that survived to discharge with a median survival time of 792.2 days (range 39–2002).

#### 3.5.1. Cause of the Effusion

The cause of the effusion was significantly associated with survival to discharge *(p <* 0.001). Excluding categories with fewer than 10 cases, uroperitoneum cases had the highest percentage of survival to discharge at 77% with an odds ratio (OR) of 2.87 and a 95% confidence interval (CI) of 1.38–5.98, and cases with volume overload and hemoperitoneum cases had the lowest percentage of cases that survived to discharge (30%, OR 0.33 and CL 1.12–3.12 and 40%, OR 0.51 and 95% CI of 0.254–1.01, respectively) (Table 5). Six of the uroperitoneum cases were managed conservatively, with one case dying before a planned exploratory laparotomy. The remaining 36 cases were managed surgically. Surgical management was significantly associated with an improved survival to discharge (*p =* 0.04) with 30/36 cases who underwent exploratory laparotomy alive at discharge.

When cases with a uroperitoneum were removed from survival analysis, 53% survived to discharge (243/455) vs. 55% when uroperitoneum cases were included. When cats diagnosed with FIP were removed from the survival analysis, 57% of cases (247/429) survived to discharge.

#### 3.5.2. Signalment

Pedigree cats were less likely to survive to discharge compared to non-pedigree cats (48% and 55%, respectively, *p* = 0.035). After removing FIP cases, no significant difference in survival to discharge was seen between pedigree and non-pedigree cats *(p =* 0.111). Age was not significantly associated with survival *(p =* 0.285). This was also true after removing FIP cases *(p =* 0.951). Sex did not affect survival to discharge with or without the FIP cases included *(p =* 0.631 and *p* = 0.466, respectively).

#### 3.5.3. Imaging Findings

The subjective measurement of effusion volume was significantly associated with survival to discharge *(p =* 0.012). A larger proportion (64%) of cats with a mild effusion survived to discharge compared to cats with a moderate effusion (51%) and cats with a marked effusion (49%). The presence of a concurrent pleural effusion did not significantly impact survival to discharge (48% with pleural effusion vs. 58% without pleural effusion survived to discharge, *p* = 0.117). This was also true when FIP cases were removed from the analysis *(p =* 0.614).

#### 3.5.4. Type of Effusion and Diagnostic Test Results

Using the χ^2^ test, the type of effusion based on protein and cellular concentrations was not associated with survival to discharge *(p =* 0.361). Looking at effusion TNCC and TP separately, neither was associated with survival to discharge *(p =* 0.268 and *p* = 0.260, respectively). Using two-tailed t-tests for parametric and Mann–Whitney U-tests for non-parametric data, lymphocyte concentration, neutrophil concentration, PCV and albumin were all significantly associated with survival to discharge. However, on multiple logistic regression analysis, only lymphocyte concentration was found to be positively associated with survival to discharge *(p =* 0.036), as shown in Table 6. This was persistent when FIP cases were removed from the analysis *(p =* 0.027). Ten cases had a lymphocyte concentration above the reference range. Seven of these cases survived to discharge. The diagnoses of these cases varied substantially (FIP in two cases and one case each of myeloid leukemia, anuric renal failure, nephrotic syndrome, pyogranulomatous and fibrosing cholangiohepatitis, IMHA, lymphoma, lymphoplasmacytic and suppurative portal hepatitis and a splenic and hepatic hemangiosarcoma).

## 4. Discussion

This study aimed to describe the survival of cats presenting with ascites and the different etiologies of this condition as well as identifying risk factors for a worsened prognosis. The most common causes of a peritoneal effusion in the current study population were septic peritonitis (86/498), followed by neoplasia (84/498), sterile inflammatory disease (79/498) and feline infectious peritonitis (69/498). Overall, 55% of cases survived to discharge from hospital. Cases who survived to discharge from hospital had a median survival time of 30.5 days after discharge. This is similar to the 21 days previously reported [1]. However, the previous study reported cardiac disease as the most common cause of peritoneal effusions. The discrepancy in the prevalence of cardiac disease between the two studies is likely due to dietary taurine deficiency associated with dilated cardiomyopathy being prevalent in cats at the time of the previous study [14,15].

Comparing the survival to discharge of different underlying etiologies of peritoneal effusion in our study to previous research showed a similar survival to discharge in cats with septic peritonitis (45%) [16]. However, in another population of cats with septic peritonitis, survival to discharge was reported in 69% of cases [17]. This discrepancy may be due to Scotti et al. excluding cases that were euthanized prior to surgery. We also found a similar percentage of cats had septic peritonitis due to intestinal rupture (48% compared to the previously reported 49.9%) and primary septic peritonitis (22.3% compared to 22.3%) [17].

Cats with neoplasia as the primary cause of ascites were found to have a survival to discharge rate of 56% with a median survival time of 28 days after discharge. The previous literature has evaluated the survival times associated with specific neoplastic causes, including carcinomatosis (reporting that 70% of cats were euthanized on diagnosis) [18]. In our study, out of 17 cats with carcinomatosis, 9 cases survived to discharge, with a median survival time of 14 days post-discharge, comparable to the previous literature. However, due to the small case numbers in the current study, this finding should be interpreted with caution.

Only three cats with idiopathic chyloabdomen were available for analysis in the current study. One did not survive to discharge, one died 2 days post-discharge and the other died 12 days post-discharge. The small number makes it difficult to extrapolate survival times for cats with this condition; however, the incidence of idiopathic chyloabdomen in cats has previously been reported in 2 cases per 100,000, with a median survival time of 44 days from diagnosis [19].

The survival rate associated with feline spontaneous hemoperitoneum has previously been reported as 12% (8/65) of cats surviving to discharge in one study [8], while our study showed that 40% of hemoperitoneum cases (14/35) survived to discharge. This discrepancy could be due to the difference in overall case numbers. Survival rates could also be related to access to feline blood products which were readily accessible in the institution where the current study was performed. However, data on the number of cases receiving blood transfusions would be needed for each study to assess this possibility fully.

The current study showed that 77% percent of cats with uroperitoneum survived to discharge, similar to a previously reported 74% [20]. A further study reported that cats with a traumatic uroperitoneum had a survival rate of 71% surviving to discharge [21]. The number of cases that were managed conservatively or surgically was examined. However, due to the very small number that were managed conservatively, the impact of treatment on survival should be interpreted with caution.

The percentage of cats diagnosed with FIP (or with a strong suspicion of FIP) in this study was 42%. However, FIP treatment has significantly changed since the study data was collected, as the availability of Remdesvir and GS-441524 has led to a markedly improved prognosis, with >80% survival reported at 6 months [22] and 81.3% of cats reported to be alive at the end of the treatment period [23,24]. This prognosis is likely to continue improving as FIP treatment may become more affordable in the future. Although this study does not reflect this recent change in FIP treatment and prognosis, FIP remains fatal for untreated cats [25], which is relevant to cases in which FIP treatment is cost-prohibitive. Statistical analysis on the impact of variables on survival was performed with and without FIP cases to allow for how the recent change in available treatment may affect the prognosis of cats presenting with ascites.

In this study, 69% of cases were non-pedigree in comparison to the general UK population, in which ~90% of cats are reported to be non-pedigree [26,27]. This could be due to the known predisposition of some breeds to pathologies that cause an abdominal effusion, such as pedigree breeds being predisposed to FIP [28] and the predisposition of Maine Coon and Ragdoll breeds to hypertrophic cardiomyopathy [29,30]. However, even when cases with FIP and cardiac disease were removed from the analysis, the proportion of non-pedigree cats with an abdominal effusion was still lower compared to the general population (75%). This could also be due to the study population being from a referral center and may be biased towards a population with good insurance or more affluent owners who are more likely to choose pedigree breeds and present to a referral center. The lack of genetic variation in pedigree breeds may also play an important role in disease development. Male cats were also mildly over-represented in this study, at 61% of cases. Male cats have been shown to be over-represented in cases with FIP [28] and cardiomyopathies [31]. A mildly increased proportion of male to female cats also persisted when FIP and cardiac cases were removed (58%); however, this was not statistically significant and was a generalized finding in all groups of causes of ascites.

A bi-cavitary effusion was identified in 127 cases (26% of all cases), which was associated with a lower percentage of survival to discharge, although not statistically significant. Previously reported median survival times for cats with a bi-cavitary effusion is 3 days, reflecting a potentially worse prognosis compared to an abdominal effusion alone [32]. However, most cats in our study had a POCUS as the only form of thoracic imaging and the sensitivity of this imaging modality for detecting pleural effusion (previously reported at 83% in dogs [33]) may have resulted in an underdiagnosis of bi-cavitary effusion. This was likely compounded by the lack of standardization of POCUS in this study population and by the fact that this form of imaging was generally not carried out by a board-certified emergency and critical care or radiology specialist. We also identified that not all abdominal effusions were detected by POCUS, but were later identified by other imaging modalities. This suggests that some cases of abdominal effusion in the population of cats in this study may have been incorrectly excluded when a POCUS was their only form of abdominal imaging. However, some cases may have developed effusion after having had a POCUS prior to further imaging.

The subjective measurement of effusion volume was also found to be significantly related to survival, and cats with a smaller volume of effusion were found to have an increased survival to discharge. This is likely related to the more common benign causes of small-volume effusions in this study, which had a better prognosis than causes of a larger effusion, rather than the size of the effusion itself being the cause of change in prognosis. This was demonstrated by cases in the volume overload and sterile inflammatory disease groups being associated with a mild effusion compared to the groups of FIP, neoplasia and hemoperitoneum cases, which seemed more often associated with a large effusion and poorer prognosis. The volume of effusion could be useful in helping to predict the cause of ascites, subsequently aiding the prediction of the prognosis. In human medicine, the volume of ascites has also been speculated to be associated with the prognosis in individual disease processes rather than an indicator of the pathology. This has been shown in cases of pancreatitis, hepatic cirrhosis, ovarian neoplasia and patients with hepatocellular carcinomas undergoing trans-arterial chemoembolization for hepatocellular carcinomas [34,35,36,37].

A significant limitation of the assessment of volume effusion in this study is the subjective nature of the measurement, the lack of standardization in imaging modalities used to make this assessment and the lack of standardization of the personnel making this assessment, including the lack of interobserver reliability assessment. More objective analysis could be considered in the future to try and more accurately quantify ascites volume and then assess the association with the prognosis. Techniques for more objective quantitative assessment of ascites volume in people have been described both for ultrasound [38,39] and CT [35,37,40] could be developed further in small animal medicine.

The type of effusion based on cellular and protein concentrations was not significantly associated with survival, which is concordant with the previous literature [32]. Protein and cellular concentrations have also importantly been previously shown to lack concordance with the cause of peritoneal effusion in cats, and can be misleading [41]. An example of this is in a case of bile peritonitis in the current study, which was confirmed on measurement of effusion bilirubin, but the fluid was categorized as a transudate and was less inflammatory than might be suspected. Possible causes of this are the proximity to when the bile leakage occurred in relation to sampling and that significant inflammation did not have time to develop. Another possibility is that, when only small pockets of fluid can be sampled, these may not fully represent the underlying pathology. This highlights the importance of not discarding a possible diagnosis based on the protein and cytological assessment of an effusion alone, e.g., if bile peritonitis or uroabdomen are suspected.

Clinicopathological parameters including patient PCV, albumin and neutrophil concentrations were not significantly associated with survival. However, lymphocyte concentrations did seem to be related to survival to discharge. This was a surprising finding, as the cases with a lymphocyte concentration above the reference range had pathologies generally associated with a poor prognosis (e.g., acute leukemia). However, previous studies have also shown that lymphopenia can be negatively associated with survival in specific conditions, such as canine hepatocellular carcinoma [42] and a higher lymphocyte concentrations being a positive prognostic indicator in feline IMHA [43]. Leukocyte ratios were not assessed in the current study, but may account for the possible survival benefit of a higher lymphocyte concentration, having previously been assessed as possible prognostic indicators in inflammatory conditions [44,45,46]. However, this finding should continue to be interpreted with a great deal of caution and further research is needed to explore the relationship of lymphocyte concentration to survival in cats with ascites. It should also be noted that, although the higher lymphocyte concentration is correlated with survival to discharge, this did not equate to long-term prognosis and was not reflective of some patients potentially being discharged with the intention of euthanasia soon after.

This study was limited by its retrospective nature, with missing data for some cases. A lack of follow-up for all cases reduced the data available for the calculation of survival times. The number of cases that were euthanized as opposed to having a natural death is likely to have also biased survival analysis. However, this still provides important information about the causes of euthanasia, including financial constraints, concerns about welfare and prognosis which are all factors that will apply to future veterinary patients presenting with ascites. Some groups of peritoneal effusion, e.g., chyloabdomen bile peritonitis, trauma and portal hypertension, only contained a few cases each and the small group size reduced the generalizability of survival times for these conditions. A further limitation was the presumptive diagnosis in FIP cases and that not all cases had the same diagnostics performed, which may have led to a misdiagnosis of some cases. Similarly for cases of neoplasia, the diagnosis was often based on cytology rather than all cases being confirmed by histopathology. Not all cases with cardiac disease underwent sampling of the effusion, which may have led to missing other concurrent causes of ascites.

## 5. Conclusions

This study demonstrates an overall guarded prognosis for cats presenting with an abdominal effusion, regardless of the cause. It also confirms that a uroabdomen is generally associated with a better prognosis than other common causes of abdominal effusion in cats. This is important, especially in a first-opinion setting, where abdominocentesis can be performed. This is a relatively inexpensive procedure that does not require specialist skills, and which could provide significant prognostic information. Furthermore, a subjective assessment of the amount of effusion present on POCUS and abdominocentesis (particularly in the case of a moderate to marked effusion vs. minimal) arguably does not require a specialist skill set or specialist equipment as they are relatively inexpensive tests to perform. Based on the current study, both these diagnostics could provide a significant indication of prognosis for cats presenting to a first-opinion practice with ascites.

## Figures and Tables

**Table 1:** Characterization of effusions and underlying etiology. In cases where the characterization of the effusions overlapped, e.g., hemorrhagic effusions which could also be defined as a protein-rich transudate based on TNCC and TP, the effusions were defined as hemorrhagic alone.

Cause of Effusion			Type of Effusion			Total Available for Analysis
	Chylous	Exudate	Hemorrhagic	Protein-Rich Transudate	Transudate	
Bile peritonitis	0	1	0	0	1	2
Hemoperitoneum	0	0	32	0	0	32
Uroperitoneum	0	4	1	4	13	22
Chyloabdomen	1	1	0	1	0	3
Neoplastic	1	16	2	48	5	72
Cardiogenic	1	3	1	10	0	15
FIP	0	27	0	32	1	60
Septic	0	42	1	3	0	46
Undefined	0	1	0	8	1	10
Trauma	0	1	0	2	0	3
Hypoalbuminemia	0	0	0	0	3	3
Volume overload	0	0	0	5	7	12
Sterile inflammatory	0	14	1	40	17	72
Portal hypertension	0	0	0	2	1	4
Recent surgery	0	1	0	3	1	5

**Table 2:** Breakdown of diagnoses in each underlying etiology of ascites.

Etiology	Number of Cats	Final Diagnosis
Septic peritonitis	87	Undefined = 31
		Neoplasia = 21
		Post-enterotomy/enterectomy = 6
		Ulceration of the stomach or duodenum = 5
		Trauma = 4
		Hepatic abscess = 3
		Pyometra = 3
		Intestinal abscess = 2
		Necrotizing jejunitis = 2
		Bacterial cholangitis = 2
		Foreign body with perforation = 2
		Fungal = 1
		Pancreatic abscess = 1
		Subcutaneous abscess = 1
		Intussusception = 1
		Ulcerative and necro-suppurative cystitis = 1
		Cystocentesis = 1
Neoplasia	84	Lymphoma (all forms) = 29
		Undefined = 10
		Carcinomatosis = 15
		Carcinoma = 10
		Intestinal adenocarcinoma = 5
		Leukemia = 4
		Epithelial undefined = 2
		Round cell undefined = 2
		Squamous cell carcinoma = 1
		Histiocytic sarcoma = 1
		Hemangiosarcoma = 1
		Sarcoma undefined = 1
		Gastrointestinal mast cell tumor = 1
		Spindle cell undefined = 1
		Multiple myeloma = 1
Sterile inflammatory	79	Hepatobiliary disease = 24
		Pyelonephritis = 1
		Gastrointestinal disease = 15
		IMHA = 9
		Pancreatitis = 13
		Undefined = 16
FIP	69	FIP = 69
Congestive cardiac failure	44	Hypertrophic cardiomyopathy = 16
		Unclassified cardiomyopathy = 12
		Arrhythmogenic right ventricular cardiomyopathy = 5
		Restrictive cardiomyopathy = 4
		Third-degree atrioventricular block = 3
		Dilated cardiomyopathy = 2
		Pericardial effusion = 1
		Pericardial peritoneal diaphragmatic hernia = 1
Uroperitoneum	43	Trauma = 15
		Urethral obstruction (feline lower urinary tract disease or urolithiasis) = 13
		Cystocentesis = 4
		Subcutaneous ureteric bypass device complication = 3
		Cystotomy tube complications = 3
		Undefined = 2
		Ovariohysterectomy complication = 2
		Post-cystectomy for a transitional cell carcinoma = 1
		Ureteric rupture due to ureteroliths = 1
Volume overload	20	Treatment of trauma with underlying cardiac disease = 1
		Renal = 15
		Enteropathy = 1
		Multiorgan dysfunction due to burns = 1
		Hepatobiliary disease = 2
Undefined	12	Undefined = 12
Recent abdominal surgery	8	Exploratory laparotomy for a suspected or confirmed foreign body = 3
		Undefined = 2
		Ovariohysterectomy = 1
		Post-surgical placement of a subcutaneous ureteric bypass system = 1
		Body wall rupture = 1
Bile peritonitis	4	Biliary obstruction = 1
		Cholangitis = 1
		Trauma = 1
		Iatrogenic post-tru-cut hepatic biopsies = 1
Chyloabdomen	3	Idiopathic = 3
Trauma	3	Road traffic accident = 2
		Bite wounds = 1
Portal hypertension	3	Portal vein thrombosis = 1
		Hepatic arteriovenous malformation and acquired shunts = 1
		Portosystemic shunt with incomplete ligation = 1

**Table 3:** Subjective measurement of effusion size for each underlying etiology and the percentage of cases of each cause of ascites which also had a concurrent pleural effusion (out of cases that underwent some form of thoracic imaging).

	Number of Cases with Description of Effusion and If a Pleural Effusion Was Present					
Cause of Effusion	Mild	Moderate	Marked	Total	Number with Pleural Effusion vs. Thoracic Imaging	Pleural Effusion (% of Cases)
Bile peritonitis	3	0	0	3	0/0	0%
Chyloabdomen	1	0	2	3	3/3	100%
Portal hypertension	2	0	1	3	0/1	0%
Trauma	1	2	0	3	1/3	33%
Hypoalbuminemia	1	1	2	4	3/3	100%
Post-surgery	6	1	0	7	1/4	25%
Undefined	1	2	4	7	5/10	50%
Volume overload	8	9	1	18	7/15	46%
Uroperitoneum	12	8	9	29	2/37	5%
Hemoperitoneum	7	11	12	30	2/31	6%
Cardiogenic	13	10	11	34	30/44	68%
FIP	8	24	30	62	22/56	39%
Septic	34	22	16	73	10/66	15%
Neoplastic	22	23	33	78	23/63	37%
Sterile inflammatory	44	25	10	79	18/58	30%

**Table 4:** Percentage of cases alive, dead or lost to follow-up at 1-year post-discharge.

	Survival to 1 Year or More			
Underlying Effusion Etiology	Dead	Alive	Lost to Follow-Up	Total
Chyloabdomen	3	0	0	3
Portal hypertension	1	1	1	3
Traumatic	0	1	2	3
Bile peritonitis	2	0	2	4
Hypoalbuminemia	4	0	1	5
Recent surgery	1	2	5	8
Volume overload	15	2	3	20
Hemoperitoneum	23	3	9	35
Uroperitoneum	11	7	25	43
Cardiogenic	23	6	15	44
FIP	59	0	10	69
Sterile inflammatory	44	11	24	79
Neoplastic	64	1	19	84
Septic	54	11	21	86
Total	314	45	139	498

**Table 5:** Survival based on the underlying etiology of ascites where more than 10 cases were present for each cause.

	Hemoperitoneum	Chyloabdomen	Bile Peritonitis	Uroperitoneum	Neoplasia	Cardiac Disease	FIP	Undefined	Septic Peritonitis	Volume Overload	Sterile Inflammatory
Survived to discharge	14/35	2/3	3/4	33/43	47/84	29/44	29/69	6/12	40/87	6/20	53/78
*p* value and OR of survival compared to all other causes with a 95% (CI)	0.51(CI 0.25–1.01), *p* = 0.057	1.61 (CI 0.14–17.09) *p =* 0.154	2.49 (CI 0.25–23.51) *p =* 0.626	2.87 (CI 1.38–5.98) *p* = 0.002	1.026 (CI 0.64–1.64) *p* = 0.9	1.62 (CI 0.84–3.10) *p* = 0.143	0.53 (CI 0.32–0.89) *p* = 0.16	0.80 (CI 0.35–2.516) *p* = 0.70	0.63 (CI 0.39–1.00) *p* = 0.029	0.33 (Cl 0.12–0.87) *p* = 0.02	1.87 (Cl 1.12–3.12) *p* = 0.015
Median survival time post-discharge where data available (days)	281	7	349	283	28	33	5	8	146	357	27.5

**Table 6:** Shows a summary of logistic regression variables and odds ratios.

	Number of Cases with Available Data	Median	Range	Reference Range	*p* Value	Odds Ratio
Fluid protein g/L	369	33	0.14–129	NA	0.640	0.995 (CI 0.973–1.017)
TNCC × 10^9^/L	338	2	0–453	NA	0.544	1.002 (CI 0.995–1.010)
Lymphocytes × 10^9^/L	275	0.95	0–24.87	1.50–6.0	0.036	1.291 (CI 1.016–1.640)
Neutrophils × 10^9^/L	302	13.73	0.05–70.2	2.5–12.5	0.690	1.006 (CI 0.976–1.038)
PCV%	392	25	2.0–61.0	25–45	0.710	1.007 (CI 0.973–1.042)
Albumin g/L	317	23.1	9.7–44.7	28–42	0.209	1.034 (CI 0.982–1.089)
Globulin g/L	284	33.5	12.6–135.7	25–46	0.152	1.021 (CI 0.993–1.050)

## Data Availability

The original contributions presented in this study are included in the article. Further inquiries can be directed to the corresponding author.

## Abbreviations

The following abbreviations are used in this manuscript:

Cl	Confidence interval
FIP	Feline infectious peritonitis
OR	Odds ratio
PCV	Packed cell volume
POCUS	Point-of-care ultrasound
TNCC	Total nucleated cell count
TP	Total protein
